# Longitudinal Assessment of Biomarkers in ALS: Discriminative Biomarkers for Disease Progression and Survival

**DOI:** 10.1002/acn3.70381

**Published:** 2026-04-06

**Authors:** David R. Beers, Yueh‐Yun Lin, Jason R. Thonhoff, Aaron D. Thome, Alireza Faridar, Weihua Zhao, Shixiang Wen, Stanley H. Appel

**Affiliations:** ^1^ Peggy and Gary Edwards ALS Laboratory, Stanley H. Appel Department of Neurology, Houston Methodist Neurological Institute, Houston Methodist Research Institute Houston Methodist Hospital Houston Texas USA; ^2^ Center for Health Data Science and Analytics Houston Methodist Hospital Houston Texas USA

**Keywords:** amyotrophic lateral sclerosis, biomarker, discriminative

## Abstract

**Objective:**

To assess the association and discriminative performance of serum biomarkers with clinical disease progression and survival in patients with amyotrophic lateral sclerosis (ALS).

**Methods:**

This retrospective study, conducted at Houston Methodist Hospital, Houston, TX, used longitudinal serum samples collected between January 2018 and December 2022. A cohort of 100 patients with sporadic or familial ALS was randomly selected and assayed by ELISAs for biomarkers 4‐hydroxy‐2‐nonenal (4‐HNE), lipopolysaccharide binding protein (LBP), and neurofilament light chain (NfL) levels.

**Results:**

Each biomarker was increased in patients. 4‐HNE and LBP were increased at diagnosis and continued to increase as the disease progressed; both correlated with progression rates and survival. NfL was increased at diagnosis, then plateaued relatively. LBP correlated with ALSFRS‐R at diagnosis; NfL did not correlate. 4‐HNE and LBP were increased in bulbar onset patients who survived a shorter period of time; NfL levels for bulbar/limb onsets were not different. Receiver operating characteristic analyses with apparent and optimism‐adjusted area‐under‐the‐curve (AUC) demonstrated that 4‐HNE and LBP discriminated rapid progression and survival, whereas NfL showed modest discrimination for rapid progression. The combination of biomarkers yielded improved AUCs as depicted in Venn diagrams across individual and combined biomarkers.

**Interpretation:**

4‐HNE, LBP, and NfL are biomarkers of lipid peroxidation, systemic inflammation, and axonal integrity. 4‐HNE and LBP correlated with disease burden, disease progression, and survival. In the bulbar onset, survival was shortened and associated with increased 4‐HNE and LBP. This exploratory longitudinal study suggests the utility of combining biomarkers to discriminate disease progression and survival and monitor clinical trial outcomes.

## 1 Introduction

Recent studies suggest that amyotrophic lateral sclerosis (ALS) is a multifactorial, multisystem disease where acute phase protein (APP), dysregulated lipid peroxidation (LP), and the decomposition of axonal structural integrities play important roles in the pathophysiology of the disease. The identification of biomarkers that address these alterations is a major area of research in ALS. These altered pathways provide the potential for biomarkers to discriminate disease burden, progression, and survival, and to reflect target engagement and treatment effects. The first reaction of the body to immunological stress is an innate, nonspecific acute phase response (APR) [1, 2]. Within hours, protein synthesis by the liver is altered, resulting in increased APPs, i.e., lipopolysaccharide binding protein (LBP); hepatocytes synthesize and secrete LBP [2, 3, 4, 5]. In addition, uncontrolled oxidative stress (OS) and the concomitant loss of free radical scavenging inflict damage to lipids; dysregulated mitochondria with the resultant production of reactive oxygen species (ROS) lead to LP [6, 7, 8, 9]. A major product of LP is the toxic 4‐hydroxy‐2‐nonenal (4‐HNE) [10, 11, 12]. Because of the decomposition of axonal integrities, neurofilament light chain (NfL) has emerged as a biomarker candidate to evaluate and quantify this decomposition; serum NfL is elevated at early stages of ALS and then plateaus over time, with higher levels discriminating shorter survival times [13, 14, 15]. Thus, OS and LP may promote axonal injury in ALS. Collectively, such data suggest that the interacting pathways of mitochondrial‐induced OS, liver‐expressed APPs, and peripheral inflammation contribute to the pathogenesis of ALS; differing levels of OS and APPs may contribute to differing burdens of disease and rates of disease progression throughout the course of ALS [16, 17, 18]. The current investigation was designed to evaluate the correlation of 4‐HNE, LBP, and NfL with disease burden and progression, and to assess their ability, individually and in combination, to discriminate progression rate and survival.

## 2 Methods

### 2.1 Study Population

#### 2.1.1 Cross Sectional Study

Standard protocol approvals, registrations, and written informed consents were obtained from all participants. All studies were performed using ethical principles for medical research involving human subjects in accordance with the Declaration of Helsinki. Patients with suspected ALS and healthy controls (HC) were recruited from Houston Methodist Hospital's (HMH) MDA/ALSA ALS clinic, patients with Parkinson's disease (PD)/HC were recruited from HMH neurology clinic, and patients with mild cognitive impairment (MCI) and Alzheimer's disease (AD)/HC were recruited from HMH's Nantz National Alzheimer Center and diagnosed by board‐certified neurologists in motoneuron disorders, movement disorders, and dementia (Table 1). Patients with ALS were diagnosed according to the revised El Escorial criteria and graded with the ALSFRS‐R scale at each clinical visit; the ALSFRS‐R was not treated as a linear decline, but a sigmoidal distribution [14, 19, 20]. For PD and MCI/AD, disease burdens were scored using the Unified Parkinson's Disease Rating Scale and Clinical Dementia Rating at each visit, respectively.

**TABLE 1:** Demographics of patients with ALS, PD, and AD, and their respective healthy controls.

	Cross sectional studies						Longitudinal studies	
	ALS patients	Healthy controls for ALS patients	PD patients	Healthy controls for PD patients	AD patients	Healthy controls for AD patients	ALS patients	Healthy controls for ALS patients
*N*	20	11	20	20	20	20	100	100
Male/Female %/%	13/7^b^ 65%/35%	7/4 64%/35%	12/8^b^ 60%/40%	11/9 55%/45%	9/11^b^ 45%/55%	10/10 50%/50%	64/36^b^ 64%/36%	60/40 60%/40%
Age at first visit/range (years)^a^	58.8 ± 1.6^b^ 25 to 75	57.6 ± 2.2 27 to 77	71.2 ± 9.1^b^ 60 to 89	70.5 ± 2.3 63 to 78	74.2 ± 5.0^b^ 66 to 83	73.6 ± 5.1/65 to 81	63 ± 11^b^ 27 to 82	61 ± 1 5 30 to 75
Age at death/range (years)^a^	ND	—	—	—	—	—	65 ± 11/35 to 85	—
Disease duration/range (months)^a^	ND	—	—	—	—	—	33.3 ± 21.0/1.5 to 102.5	—
Disease burden	35 ± 9.9 at diagnosis ALS‐FRS‐R	—	2.6 ± 1.1 UPDRS	—	1.08 ± 0.7 CDR	—	36 ± 9.3 at diagnosis ALS‐FRS‐R	—
Limb versus bulbar onsets of disease	ND	—	—	—	—	—	65 limb/32 bulbar (3‐other sites)	—
Ethnicity (%)								
Caucasian	17	9	14	15	17	18	85	83
Black	0	0	0	0	0	0	7	6
Hispanic	2	0	0	3	2	0	6	5
Asian/other	1	2	6	2	1	2	2	6

Abbreviations: ALS‐FRS‐R = Amyotrophic Lateral Sclerosis Functional Rating Score – Revised; CDR = Clinical Dementia Rating; ND = not determined; UPDRS = Unified Parkinson's Disease Rating Scale.

^a^ Presented as mean ± SD.

^b^ Not significantly different from healthy controls.

#### 2.1.2 Longitudinal Study

This was a retrospective study of patients with ALS and HC from HMH's MDA/ALSA ALS clinic. Patients undergo detailed pulmonary function, nutritional assessments, and neuropsychological testing. All patients provided detailed family histories and were tested for common genetic mutations, i.e., superoxide dismutase 1 (SOD1), C9orf72, TDP43, etc. All patients with ALS received standard care.

Survival was calculated from the date of diagnosis to the date of death, and dichotomized at the mean, where survival months less than 33.33 were considered poor survival. Rapidly progressing patients were defined as those progressing at a rate of greater than or equal to 1 ALSFRS‐R point/month, whereas slowly progressing patients progressed at less than 1 ALSFRS‐R points/month [8]. HC were typically spouses and friends of patients, and exclusion criteria included any neurologic condition, autoimmune diseases, or infectious diseases. Clinical information was collected by the investigator from symptom onset and diagnosis to baseline assessment and sample collection.

This study used immediately frozen longitudinal sera samples from 352 patients collected between January 2018 and December 2022. From this repository, a cohort of 100 patients was selected using a computer‐based random sampling generator for exploratory analysis. Of these 100 patients, 3 patients had C9orf72 mutations, and 2 patients had a family history of ALS, thus 5%. Baseline demographics of this 100‐patient cohort are shown in Table 1. Four to eleven longitudinal sera samples were available per patient, with a mean of 6 samples/patient. All patient sera samples included blood samples drawn at diagnosis, then throughout the course of disease, with the last blood drawn at/near end‐stage disease.

### 2.2 Sample Collection, Processing, and Storage

For serum analyses, blood was collected in a red top BD vacutainer (BD, Franklin Lakes, NJ) and allowed to clot upright at room temperature for 1 to 2 h. Following centrifugation (1750 g for 10 min at 4°C) serum was aliquoted into cryogenic sterile freestanding conical microtubes (Nalgene [Nalge Nunc International Corporation, Rochester, NY]) and quickly stored at −80°C until use. For this cohort of patients with ALS, longitudinal sera samples from each patient were assayed for 4‐HNE, LBP, and NfL levels by standard ELISAs, and the values were compared with clinical outcomes at each blood draw. The 4‐HNE, LBP, and NfL levels from sera samples of 100 age‐ and gender‐matched HC were also collected; HC samples were repeated to ensure that several freeze/thaw cycles did not alter baseline levels of each biomarker.

### 2.3 Assessing Biological Markers of Lipid Peroxidation, Inflammation, and Axon Structural Products

To assess LP, 4‐HNE levels were assayed in sera of patients and HC by ELISA according to the manufacturer's instructions (Cell Biolabs Inc., San Diego, CA, USA—catalog number STA‐838). A commercially available LBP ELISA (Abnova—catalog number KA0448) was used to quantify the inflammatory serum LBP levels in patients and HC (calibration range: 1.5 to 50 ng/mL). Serum NfL concentrations were quantified by Quanterix using the Uman Diagnostics NfL ELISA kit (Quanterix (Uman) ELISA kit: catalog number 20–8002; detection limit: 33 pg/mL; calibration range: 100 to 10,000 pg/mL). Samples were diluted to fall within the range of the standard curves for 4‐HNE, LBP, and NfL. None of the assays fell below detectable limits.

### 2.4 Statistical Evaluations

Analysis of Variance (ANOVA) was utilized to compare group differences across more than two groups, and Student's *t*‐test was performed for comparisons between two groups when the data were normally distributed, along with the corresponding *p‐*value. For non‐normally distributed data, the Kruskal–Wallis test was used to compare group differences across more than two groups, while the Mann–Whitney *U* test was performed for two‐group comparisons. Normality was assessed using the Shapiro–Wilk test (results omitted). Power analyses determined that this biomarker study was adequately powered.

Receiver operating characteristic curve (ROC) analyses were performed to assess the discriminative performance of individual and combined biomarkers (4‐HNE, LBP, and NfL) for poor survival and rapid progression. ROC curves and the corresponding apparent area‐under‐the‐curves (AUC) were constructed for each biomarker and biomarker combination. The differences across AUC were then tested using nonparametric DeLong's test. Internal validation was performed using bootstrap resampling with 2000 iterations, and optimism‐adjusted AUCs were calculated by subtracting mean optimism from the apparent AUCs of the ROC analyses. The progression rate was calculated by the changes in the ALS‐FRS‐R score per survival month.

## 3 Results

### 3.1 Cross‐Sectional Studies of 4‐HNE, LBP, and NfL as Biomarkers in ALS, PD, and AD

4‐HNE, a biomarker of oxidative stress; LBP, a biomarker of systemic inflammation; and NfL, a biomarker of axonal injury, were assessed in cross‐sectional analyses of rapidly and slowly progressing patients with ALS, patients with PD, and patients with MCI and AD (Table 1). 4‐HNE was higher in rapidly progressing patients with ALS compared with HC (*p* < 0.001) (Figure 1A), as well as in rapidly progressing patients compared with slowly progressing patients (*p* = 0.003); no 4‐HNE difference was found in slowly progressing patients compared with HC. In addition, no 4‐HNE differences were found among patients with PD, MCI or AD (Figure 2A,D).

**FIGURE 1:**
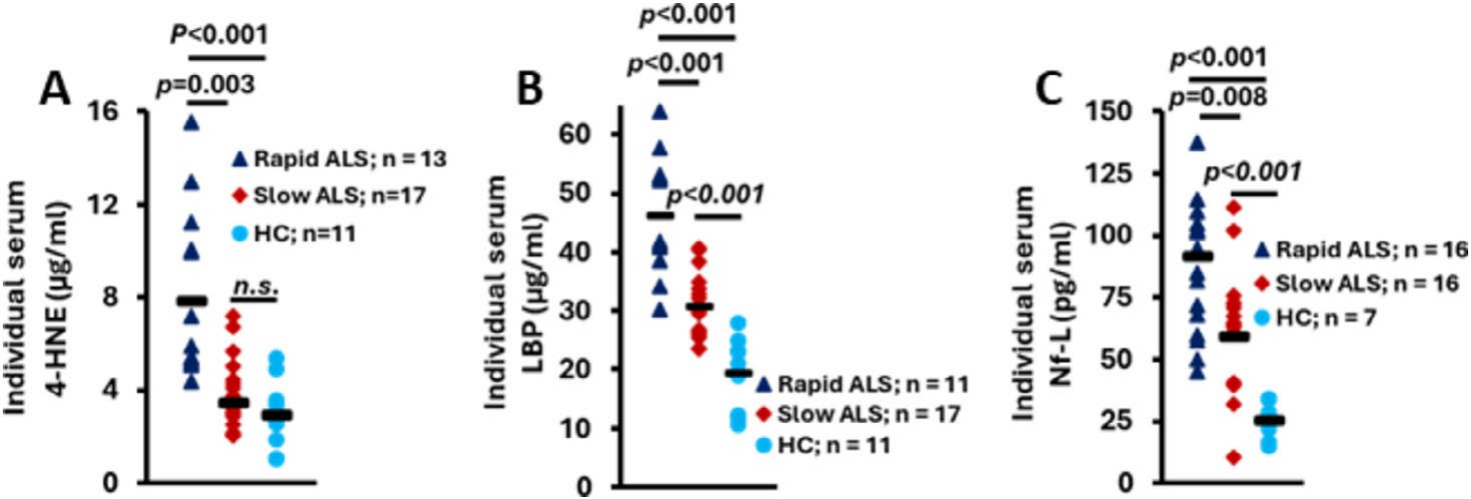
Cross‐sectional assessments of the 4‐HNE, LBP, and NfL as biomarkers in the sera of patients and HC. (A, B, C) Serum level of 4‐HNE, LBP, and NfL in rapidly and slowly progressing ALS patients, respectively. These data suggest 4‐HNE, LBP and NfL are collectively elevated in patients with ALS compared with HC. Statistical comparisons were performed as described in the Methods with corresponding *p*‐values.

**FIGURE 2:**
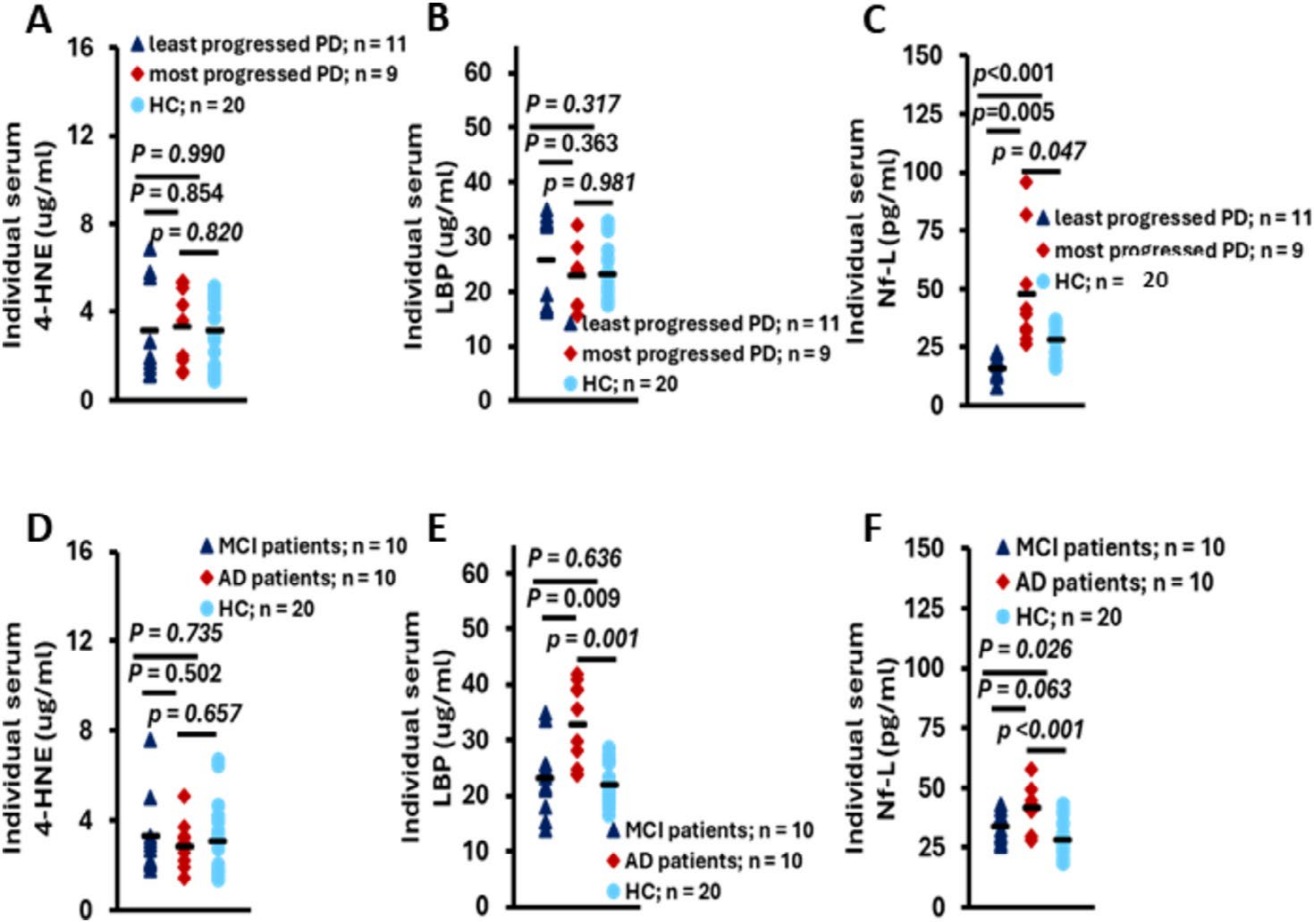
Cross‐sectional assessments of the 4‐HNE, LBP, and NfL as biomarkers in the sera of patients with PD, MCI, AD, and compared with HC. (A, B, C) Serum level of 4‐HNE, LBP, and NfL, in the least and most progressed PD patients, respectively. (G, H, I) Serum levels of 4‐HNE, LBP, and NfL in MCI and AD patients, respectively. These data suggest 4‐HNE, LBP and NfL are collectively elevated in patients with ALS compared with PD, MCI/AD patients, and HC. Statistical comparisons were performed as described in the Methods with corresponding *p*‐values.

LBP was different across ALS progression groups, with LBP higher in rapidly progressing patients compared with slowly progressing patients and HC (both *p* < 0.001) (Figure 1B). LBP was also higher in slowly progressing patients compared with HC (*p* < 0.001). No difference was found between patients with PD and HC (Figure 2B). However, LBP was higher in patients with AD compared with patients with MCI (*p* = 0.009) and HC (*p* = 0.001) (Figure 2E).

Differences were found in NfL across disease groups. NfL was higher in rapidly progressing patients with ALS compared with slowly progressing patients (*p* = 0.008) as well as HC (*p* < 0.001) (Figure 1C). NfL was also higher in slowly progressing patients compared with HC (*p* < 0.001). Among the patients with PD, NfL was increased in the most progressed patients compared with the least progressed patients (*p* = 0.005) as well as HC (*p* = 0.047) (Figure 2C). In contrast, serum NfL was lower in the least progressed patients with PD compared with HC (*p* < 0.001). NfL was higher in patients with AD compared with HC (*p* < 0.001), but not compared with patients with MCI (Figure 2F). Patients with MCI had higher levels of NfL compared with HC (*p* = 0.026).

### 3.2 Longitudinal Correlations of 4‐HNE, LBP, and NfL as Biomarkers for Survival and Rates of Progression

Levels of 4‐HNE, LBP, and NfL at diagnosis were compared with HC. At diagnosis, mean levels of NfL were increased to the greatest extent, approximately 3.3‐fold, while levels of LBP were elevated compared with HC (approximately 1.6‐fold). 4‐HNE was modestly increased compared with HC, approximately 1.3‐fold (Figure 2A–C). However, as a measure of the extent of disease, from diagnosis to last sample, 4‐HNE had the greatest relative increase (177%) followed by LBP (80%) (Figure 3D). Despite having the highest levels at diagnosis, NfL increased only modestly (27%) from diagnosis to the last sample, indicating a plateau of the biomarker.

**FIGURE 3:**
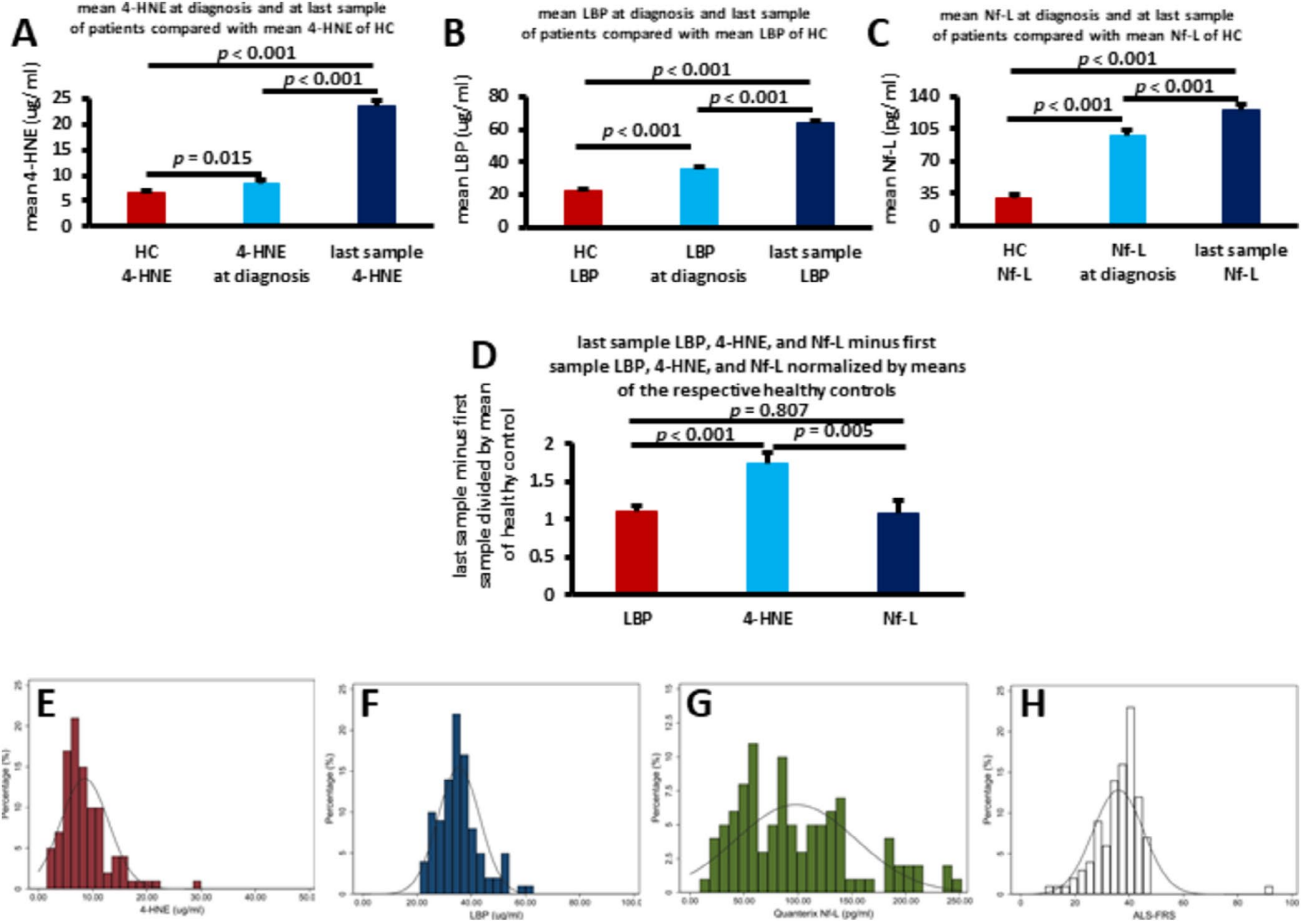
Comparison of mean biomarkers 4‐HNE, LBP, and NfL in the sera of longitudinally studied patients and their respective normalization curves. (A, B, C) Mean serum levels of 4‐HNE, LBP, and NfL, in patients at diagnosis and at the last available sample. (D) Changes in serum levels of 4‐HNE, LBP, and NfL at diagnosis to the last sample, normalized to 1. (E, F, G) Normalization curve of 4‐HNE, LBP, and NfL in the sera of patients. (H) Normalization curve of the patients with ALS ALSFRS‐R scores. *p* = *t*‐test.

The distributions of the 4‐HNE, LBP, and NfL levels were depicted as histograms with overlayed normality curves (Figure 3E–G). All three biomarkers were non‐normally distributed, with median (IQR) of 4‐HNE, LBP, and NfL as 7.57 μg/mL (5.69), 35.03 μg/mL (31.01), and 88.42 pg/mL (55.39), respectively. For clinical measurements, survival months were also non‐normally distributed (median = 27.50 months, IQR = 15.75), whereas progression rate was normally distributed with a mean of 1.09 ALSFRS‐R points/month (SD = 0.54). The ALSFRS‐R percentages also showed a nonnormal distribution (median = 37.00, IQR = 9.00) (Figure 3H).

Next, each biomarker at diagnosis was assessed for its correlation to the patient's ALSFRS‐R score at diagnosis. There was a weak correlation between 4‐HNE and the ALSFRS‐R score (*r* = −0.274; *p* < 0.001) (Figure 4A). LBP showed the strongest correlation with the ALSFRS‐R score (*r* = −0.688; *p* < 0.001) (Figure 4B). In contrast, no serum NfL level correlation was found with patients' ALSFRS‐R scores (Figure 4C). Additionally, each biomarker was correlated with the others, with a moderate correlation between LBP and 4‐HNE (*r* = 0.551, *p* < 0.001) and NfL and 4‐HNE (*r* = 0.470, *p* = 0.001) (Figure 4D,E). However, only a weak correlation between NfL and LBP was identified (*r* = 0.289, *p* < 0.001) (Figure 4F).

**FIGURE 4:**
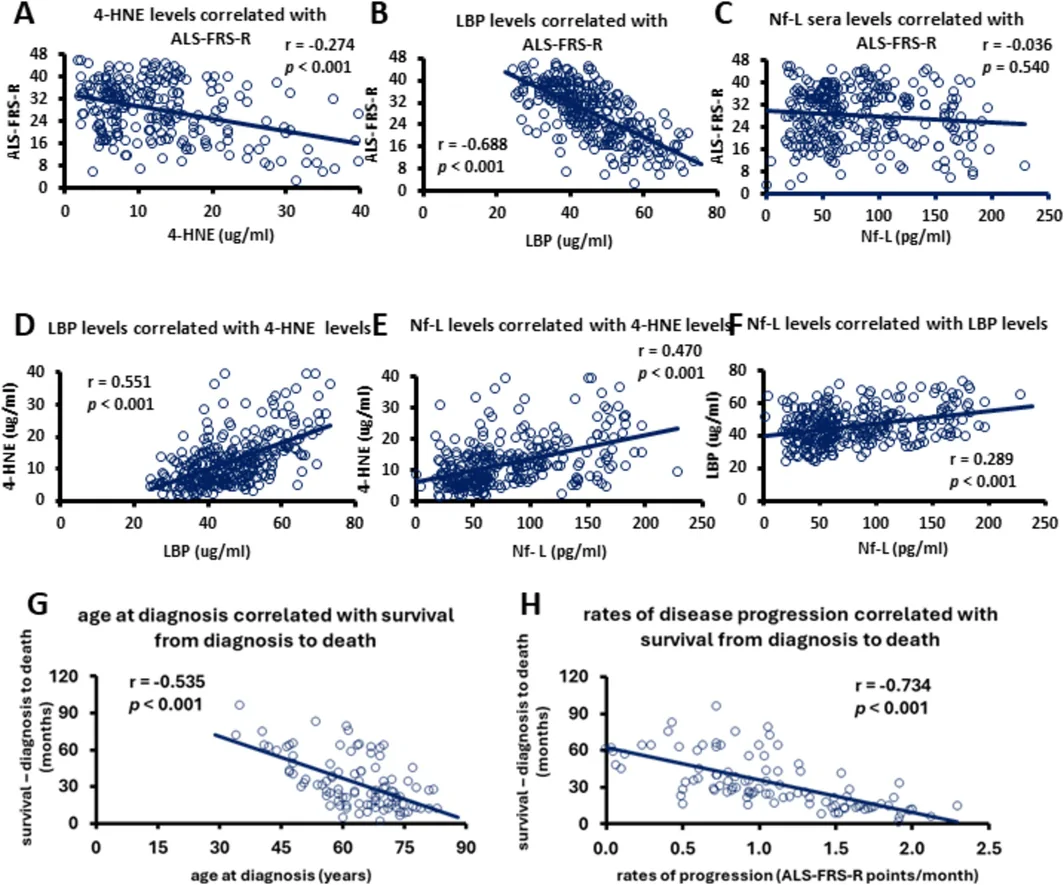
Correlation assays among biomarkers (4‐HNE, LBP, and NfL), ALSFRS‐R scores, age, and progression rate in the sera of longitudinally studied patients at each sample. (A, B, C) The correlation between 4‐HNE, LBP, and NfL sera levels, respectively, and the corresponding ALSFRS‐R scores. (D, E, F) Pairwise correlations between 4‐HNE, LBP, and NfL sera levels. (G) Correlation between age at diagnosis and Survival in patients with ALS. (H) Correlation between rate of disease progression and Survival from diagnosis to death in patients with ALS. Correlation coefficient is reported as *r* = Spearman's rho value with the corresponding *p* values.

There was a correlation between age at diagnosis and survival from the date of diagnosis to the date of death (*r* = −0.535, *p* < 0.001) (Figure 4G). Similarly, there was a correlation between rates of disease progression and survival from diagnosis to death (*r* = −0.734, *p* < 0.001) (Figure 4H). The age at disease onset showed a weak correlation with survival from onset of disease to death, suggesting the less‐than‐precise estimate of disease onset obtained by clinical history (data not shown).

All three biomarkers, 4‐HNE, LBP, and NfL, were correlated with the length of survival from diagnosis to death. 4‐HNE levels at diagnosis were correlated with survival (*r* = −0.661, *p* < 0.001) (Figure 5A). LBP showed the strongest correlation with length of survival (*r* = −0.773, *p* < 0.001) (Figure 5B). NfL levels at diagnosis were also correlated with length of survival, but the correlation was weaker than that of 4‐HNE or LBP (*r* = −0.325, *p* = 0.001) (Figure 5C).

**FIGURE 5:**
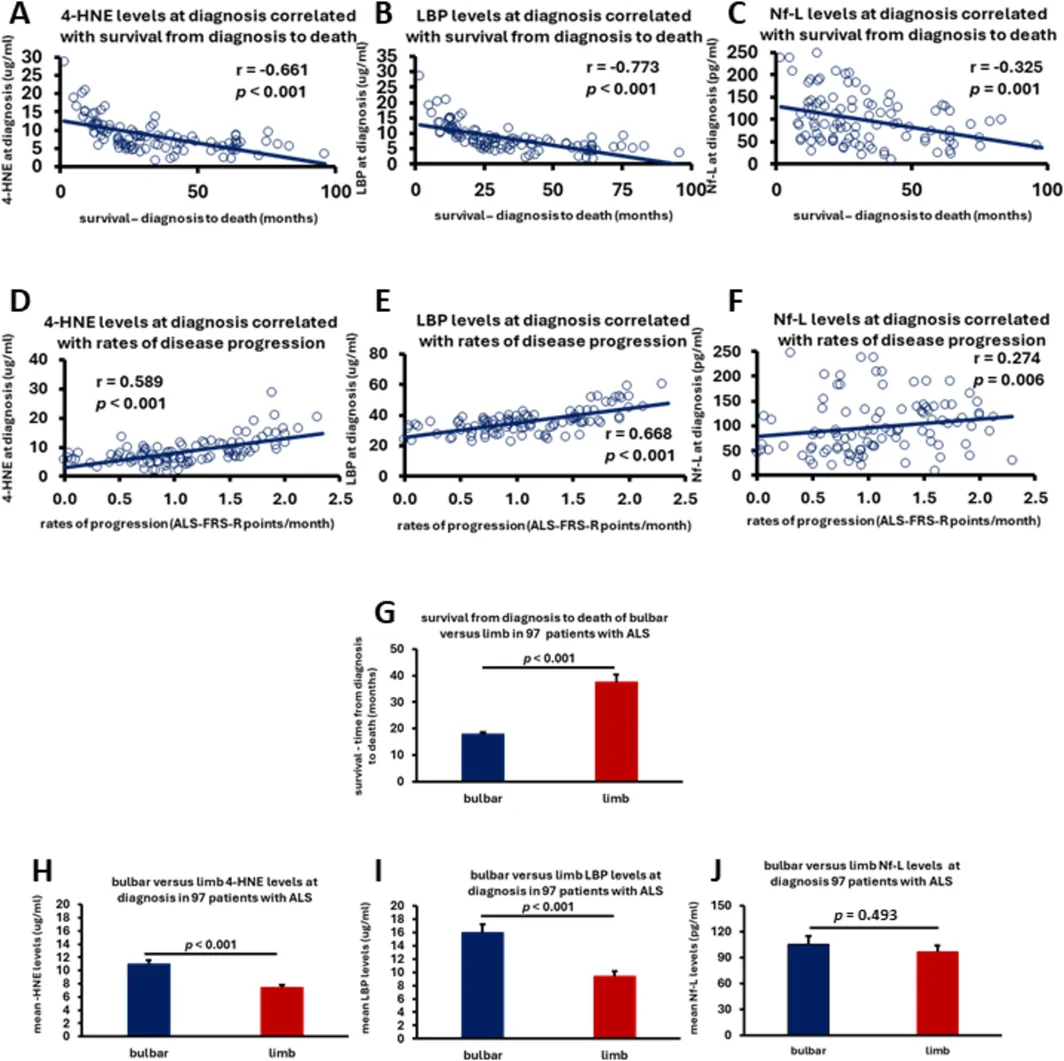
Correlations assays among biomarkers (4‐HNE, LBP, and NfL) at diagnosis with survival, rates of disease progression, and site of disease onsets in ALS patients. (A, B, C) The correlation between 4‐HNE, LBP, and NfL sera levels, respectively, and patients' survival from diagnosis to death. (D, E, F) The correlation between 4‐HNE, LBP, and NfL sera levels, respectively, and patients' rate of disease progression. (G) Comparison between 97 patients with bulbar (32) and limb (65, including arms/legs) onsets of ALS from diagnosis to death. (H, I, J) 4‐HNE, LBP, NfL, respectively, in the sera of bulbar onset ALS versus limb onset ALS. Correlation coefficient is reported as *r* = Spearman's rho value with the corresponding *p* values.

The biomarkers of oxidative stress, inflammation, and axonal integrity were evaluated for their correlation with rates of disease progression. Levels of 4‐HNE were correlated with the rate of disease progression (*r* = 0.589, *p* < 0.001) (Figure 5D). Consistent with its correlation to survival, LBP showed the strongest correlation with progression rate (*r* = 0.668, *p* < 0.001) (Figure 5E), whereas NfL demonstrated only a weak correlation with rates of disease progression (*r* = 0.274, *p* = 0.006) (Figure 5F).

Comparisons across each biomarker level were also assessed between patients with bulbar versus limb onset of disease. Of the 100 patients with ALS studied longitudinally, 97 had bulbar/limb (32 bulbar/65 limb) onsets of disease and 3 had other sites of disease onset. Among the 97 patients, patients with bulbar onset had shorter length of survival than those with limb onset (*p* < 0.001) (Figure 5G). Furthermore, the mean 4‐HNE and LBP levels were higher in patients with bulbar versus limb onsets of disease (4‐HNE: approximately 1.5‐fold, *p* < 0.001; LBP: 1.7‐fold, *p* < 0.001) (Figure 5H,I). On the other hand, levels of NfL did not differ between patients with bulbar and limb onset (Figure 5J).

### 3.3 ROC Analyses

To evaluate the discriminative performance of the 3 biomarkers (4‐HNE, LBP, and NfL) in survival and progression rates individually and in combination, ROC analyses were performed. For discriminations of poor survival, 4‐HNE and LBP showed good performance with AUC values of 0.78 and 0.75, respectively (Figure 6A and Supporting Information), and NfL reported a modest AUC of 0.66. Moreover, discrimination performance was improved when biomarkers were combined. The combination of 4‐HNE and LBP, 4‐HNE and NfL, as well as LBP and NfL, yielded higher AUCs of 0.84, 0.80, and 0.79, respectively. The highest discrimination performance was found when all three biomarkers were combined (AUC = 0.86, *p* < 0.001). These discriminative values were repeated with 3‐year survival times (Figure 6B).

**FIGURE 6:**
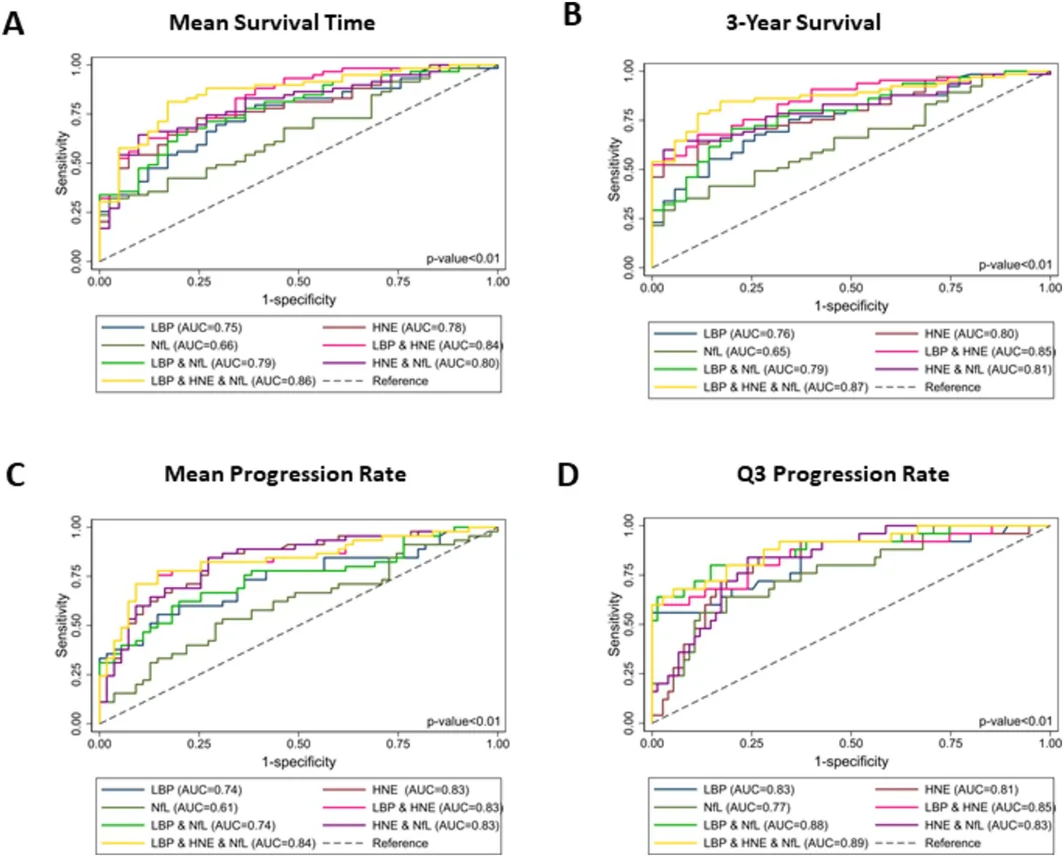
ROC analyses of the individual and combined biomarkers (4‐HNE, LBP, and NfL) for dichotomized survival and rates of disease progression of patients with ALS from all stages of disease at the time of serum collection. The post hoc discriminative values of 4‐HNE, LBP, and NfL were determined using ROC analyses. (A) ROC curves for the discriminative performance of mean survival. (B) ROC curves for the discriminative performance of 3‐year survival. (C) ROC curves for the discriminative performance of mean disease progression rate. (D) ROC curves for the discriminative performance of Q3 rates of disease progression. Apparent AUC estimates are reported with corresponding *p‐*values.

For discrimination of rapid progression, 4‐HNE and LBP demonstrated good performance with AUCs of 0.83 and 0.74, respectively (Figure 6C), and NfL reported a modest AUC of 0.61. Consistent with the survival outcome, combining biomarkers improved discriminative performance, with 4‐HNE and LBP, 4‐HNE and NfL, as well as LBP and NfL yielding higher AUCs of 0.83, 0.83, and 0.74, respectively. Again, when combining all three biomarkers, the highest discriminative performance was yielded with an AUC of 0.84 (*p* < 0.001). ROC analyses of Q3 progression rates discriminated rapid progression for the 4‐HNE, LBP, and NfL yielding AUCs of 0.81, 0.83, and 0.77, respectively (Figure 6D). The combination of 4‐HNE and LBP, 4‐HNE and NfL, as well as LBP and NfL, yielded higher AUCs of 0.85, 0.83, and 0.88, respectively. The highest discrimination performance was found when all three biomarkers were combined (AUC = 0.86, *p* < 0.001). Venn diagrams were used to depict the discriminative performance of the individual and combined biomarkers for both survival and rates of disease progression (Figure 7B,C).

**FIGURE 7:**
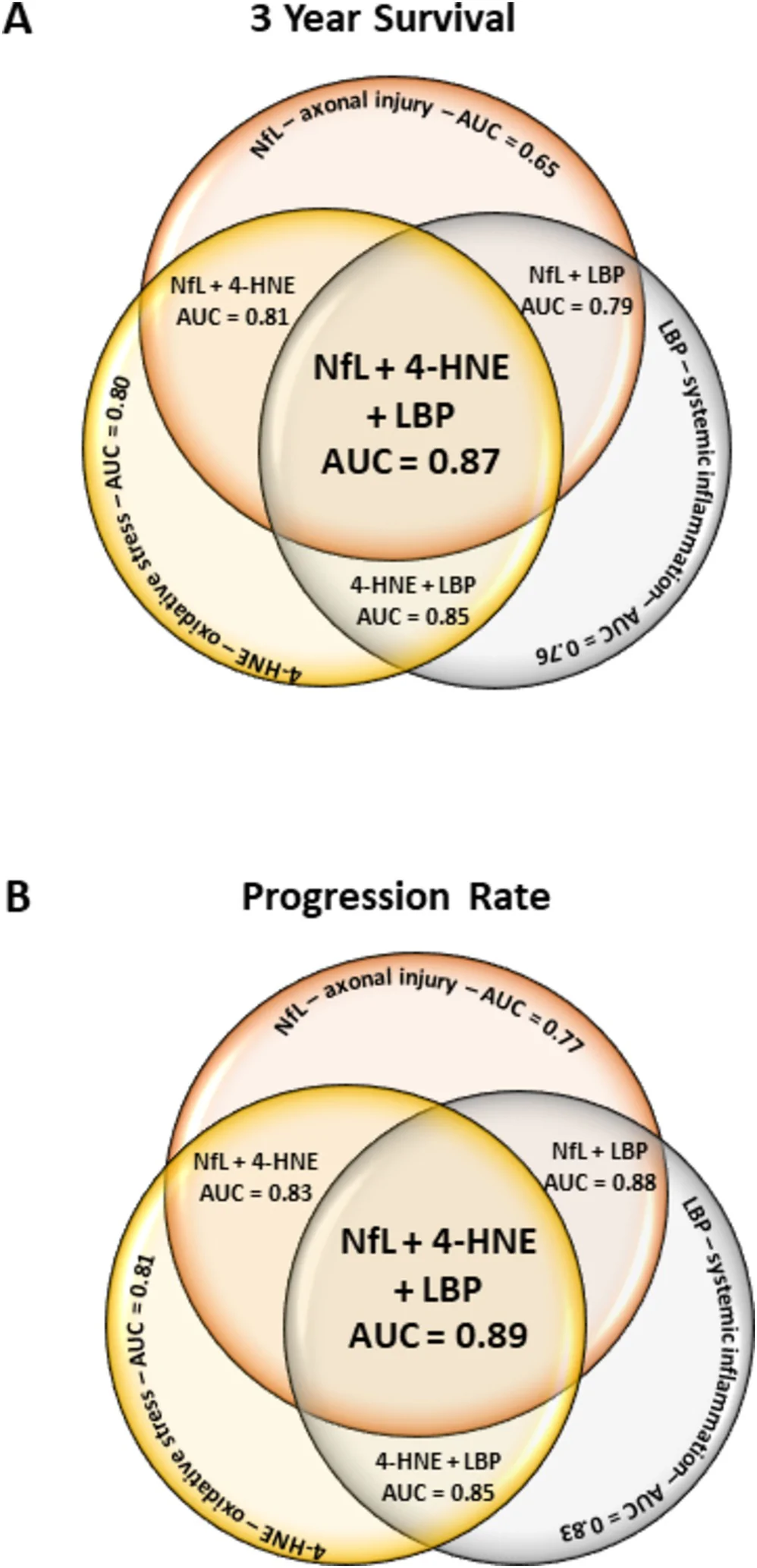
Venn diagrams including apparent AUCs across the individual and biomarker combinations of 4‐HNE, LBP, and NfL. (A) Apparent AUCs for dichotomized survival across the individual and combined biomarkers. (B) Apparent AUCs for the dichotomized rate of disease progression across the individual and combined biomarkers.

Internal validation of the ROC analyses was conducted using bootstrap resampling. Across individual and combined biomarkers, small mean optimism estimates (< 0.02) and similar optimism‐adjusted AUCs to the apparent AUCs were identified. Specifically, the adjusted AUCs for poor survival were 0.79, 0.75, and 0.66 for 4‐HNE, LBP, and NfL, respectively (Supporting Information). For rapid progression, the adjusted AUCs were 0.83, 0.74, and 0.60 for 4‐HNE, LBP, and NfL, respectively. Thus, bootstrapping resampling of the original cohort corroborated the primary ROC analyses' discriminative performances of the 3 biomarkers.

Consistent with findings based on apparent AUCs, combining biomarkers improved the discriminative performance after adjusting for optimism. For survival discrimination, combining two biomarkers yielded adjusted AUCs ranging from 0.78–0.80, with the highest optimism‐adjusted AUC identified when all three biomarkers were combined (adjusted AUC = 0.85). For rapid progression, combining biomarkers yielded adjusted AUCs ranging from 0.73 to 0.83, with the highest adjusted AUC identified for 4‐HNE, which was similar to its apparent AUC.

## 4 Discussion

Systemic inflammation is a prominent component in ALS pathobiology with both adaptive and innate immune systems contributing to disease progression and survival [21, 22, 23, 24, 25, 26, 27, 28]. Previous studies have demonstrated that as disease progresses and disease burden escalates, there is a concomitantly escalating cascade of pro‐inflammatory responses, OS, as well as axonal injury; OS induces further inflammation and continual axonal degradation [5, 21, 28]. The current investigation was designed to assess the biomarkers 4‐HNE, LBP, and NfL in patients longitudinally followed from diagnosis to death. Although this study was exploratory, previous reports have demonstrated the importance of increased levels of 4‐HNE, LBP, and NfL in the blood of patients with ALS, thus documenting the practical importance of measuring these 3 biomarkers and realistic clinical prospect of using them through routine diagnostic laboratory processes [2, 5, 6, 13, 14].

4‐HNE, LBP, and NfL were all increased in cross‐sectional analyses of fast progressing patients. NfL serum levels were elevated early and then plateaued (27%) throughout the course of disease as previously documented (Kläppe et al., reported that NfL decreased in longitudinally studied patients with ALS after diagnosis) [29, 30]. NfL did not correlate with ALS‐FRS‐R; NfL peaked early in the course of disease while ALSFRS‐R decreased at a mean rate of −1.09 points/month. Because NfL was elevated at diagnosis and increased minimally from diagnosis to last sample, and with the ALS‐FRS‐R continuing to decline over time in these patients, this may explain why NfL levels did not correlate with the ALS‐FRS‐R (subdomains of the ALS‐FRS‐R were not analyzed with respect to NfL levels). NfL is an important marker of axonal injury prior to the conversion of pre‐symptomatic mSOD1‐mediated ALS to symptomatic ALS [13, 29, 31, 32].

When the distribution of the 4‐HNE, LBP, and NfL serum biomarker levels was assessed, all three biomarkers were found to be non‐normally distributed at diagnosis. The levels of each biomarker skewed towards lower serum levels among patients, whereas the distribution of ALSFRS‐R scores skewed towards less disease burden, suggesting that patients were diagnosed at an earlier stage of disease and thus had lower serum biomarkers. On the other hand, progression rates were normally distributed; previous studies demonstrated that progression rates of ≥ 1.0 ALSFRS‐R points/month were rapidly progressing patients, whereas progression rates of < 1.0 points/month were slowly progressing patients [7, 33].

Axonal injury is characteristic not only of ALS, but also of other neurological conditions, including PD, AD, and multiple sclerosis; different factors may initiate the axonopathy and the increased serum NfL in different diseases. In patients with ALS, the translocation of TDP‐43 and altered mRNA splicing in C9orf72 result in the appearance of cryptic exons, and proteins derived from the translation of cryptic exons are increased in the sera and CSF of asymptomatic patients [15, 34]. These cryptic proteins may contribute to the early axonal changes reflected as increased serum and CSF NfL. Regardless of what initiates the increased levels of NfL, the process does not appear to be sustained at the same rate throughout the disease course, which may suggest different pathophysiological pathways in early axonal injury and subsequent systemic inflammation [35, 36].

Increased serum levels of 4‐HNE and LBP (177% and 80% from diagnosis to last sample, respectively) suggest that increasing systemic levels of OS and inflammation contribute to disease pathophysiology. Serum levels of 4‐HNE and LBP are not increased in other neurodegenerative diseases such as PD, AD, and FTD. The rise in 4‐HNE throughout the course of disease reflects the ongoing dysregulation of mitochondria and the presence of altered calcium homeostasis, resulting in decreased respiratory chain activity, decreased ATP synthesis, and increased ROS and LP [37, 38, 39, 40]. These results emphasize the widespread OS, LP, and pro‐inflammatory systemic responses that occur in ALS, with concomitantly increased peripheral levels of APPs, i.e., sCD14, C‐reactive protein, and LBP [2].

The strong negative correlation of LBP with the ALSFRS‐R supports the contribution of systemic inflammation in ALS. LBP is synthesized by and released from the liver and binds to bacterial lipopolysaccharide (LPS) to elicit immune responses by presenting the LPS to the myeloid cell surface pattern recognition receptors CD14 and Toll‐Like receptor 4 (TLR‐4) [2]. In turn, pro‐inflammatory activation of monocyte/macrophages enhances the inflammatory milieu and promotes the synthesis of LBP.

LBP is produced primarily by hepatocytes, but also by adipose tissue and intestinal cells [3, 41]. LBP can result from systemic inflammation mediated by hepatocytes and activated macrophages, but also by gut dysbiosis. The gut microbiome has been reported to be different in ALS patients compared with controls, but the changes are different in diverse ALS populations; the gut microbiome is a source of potentially disease‐modifying bioactive metabolites that may modulate metabolic, transcriptional, and epigenetic programs and contribute to the ALS pathogenesis and other neurological disorders, including PD and AD [42, 43, 44, 45, 46].

The present study was consistent with the observation that the older the age of the patient with ALS, the shorter the survival, possibly related to the increased inflammatory milieu normally present with aging; ALS‐mediated pro‐inflammatory responses may enhance this toxic pathophysiology [47]. It is also consistent that the faster the rate of progression, the shorter the survival times, and conversely, the longer the survival times, the slower the rates of progression. As the levels of 4‐HNE (1:2.8) and LBP (1:1.8) increased from diagnosis to last sample, disease progression rates increased and survival decreased, consistent with the increasing disease burden; NfL levels weakly correlated with survival and rates of progression (1:1.3). The increased levels of 4‐HNE and LBP in bulbar versus limb onsets in ALS are consistent with this observation; increased OS and systemic inflammation lead to more rapid progression and shorter survival. NfL levels in bulbar versus limbs onsets were not different, possibly related to the lower rise of axonal injury as the disease progresses compared with OS and systemic inflammation.

The ROC analyses with subsequent internal validation using bootstrapping demonstrated that 4‐HNE, LBP, and NfL serum levels showed discriminative performance for both poor survival and rapid progression rate at the time of serum collection. After internal validation, the apparent AUCs and optimism‐adjusted AUCs were found to be similar, indicating stable performance. For both survival and progression rate, the individual biomarkers showed modest to good discrimination, with 4‐HNE and LBP yielding higher AUCs compared with NfL. This study also found improved discriminative performance when biomarkers were combined compared with individual biomarkers. For survival, the highest AUCs were achieved when all three biomarkers were combined, supporting the additional value of integrating complementary biomarkers. For rapid disease progression, combined biomarkers showed comparable improvements in discrimination. The Venn diagrams illustrate the complementary contributions of individual and combined biomarkers with consistently improved discrimination for both survival and progression rates. Together, these findings suggest the potential utility of integrating 4‐HNE, LBP, and NfL to improve discrimination of clinical ALS outcomes and highlight their complementary contributions to ALS disease pathophysiology.

There are several limitations to this exploratory study to be acknowledged. First, the study cohort was derived from a retrospective, single‐center repository and was limited to 100 patients selected from a total of 352 individuals with confirmed sporadic or familial ALS. Therefore, the findings may be less generalizable to broader ALS populations. However, the study was designed as an exploratory analysis rather than a multi‐institutional biomarker study. Second, though selection bias has been an underlying concern for retrospective study design, using a computer‐generated random sampling process to select 100 patients from the larger repository helps minimize systematic selection bias. Additionally, potential confounders, including age, sex, genetic status, site of onset, and disease duration were also taken into account in the study design. Third, ROC analyses are conducted post hoc without independent prospective cohort validation, thus raising the possible concern of overfitting with combined biomarker models. To alleviate this limitation, internal validation using bootstrap resampling was performed, demonstrating minimal optimism‐adjusted estimates and stable discriminative performance across models. Lastly, several recent studies have assessed multimodal biomarker panels in patients with ALS [48]. Chia et al. [49] used a plasma proteomics‐based biomarker panel and machine learning approaches to determine that 33 proteins were differentially expressed in patients, demonstrating that the disease process impacted skeletal muscle, nerves, and energy metabolism, and occurs years before symptoms appear. The current study, focusing on biologically informed biomarkers, determined that the combination of three biomarkers, 4‐HNE, LBP, and NfL, from three diverse pathways, lipid peroxidation, inflammation, and axonal integrity, consistently improved the discrimination of both disease progression rates and survival as well as supported practical benefits, as these three biomarkers can be readily assessed by standardized procedures in a clinical diagnostic laboratory.

The current study focused on the biologically informed roles of 4‐HNE as a biomarker of OS/LP, LBP as a biomarker of systemic inflammation, and NfL as a biomarker of axonal injury, three diverse pathophysiological pathways, that improve the discrimination of disease progression rates and survival. All three biomarkers' levels were increased in patients with ALS. The increase in 4‐HNE from diagnosis to end‐stage disease is consistent with the contribution of dysfunctional mitochondria and ferroptosis‐induced lipid peroxides to the pathophysiology of disease; similarly, increases in LBP are also consistent with the contribution of systemic inflammation. Given that LBP can be derived from hepatocytes activated by pro‐inflammatory myeloid cells as well as from intestinal cells activated by gut dysbiosis, this is again consistent with the widespread systemic inflammation that characterizes ALS. Notably, higher LBP and 4‐HNE levels were correlated with a more rapid disease progression rate, shorter survival, and lower ALSFRS‐R scores. These findings raise the question of whether these biomarkers may be useful for monitoring the therapeutic slowing of disease progression and the improvements in ALSFRS‐R scores. Plasma NfL has been validated as a biomarker through its reduction in association with toferson‐mediated slowing of disease progression in mSOD1‐mediated ALS [50]. Whether LBP and 4‐HNE can similarly serve as biomarkers for monitoring treatment response in ALS remains to be determined and highlights the need for future investigation in prospective therapeutic studies.

## Author Contributions

D.R.B. and S.H.A. contributed to the conception and design of the study, drafting the text and preparing the figures, and analysis of the data. Y.‐Y.L. performed the ROC and distribution analyses. J.R.T., A.D.T., and W.Z. contributed to the analysis of the data. S.W. contributed to the acquisition of the data. All authors read and approved the final version of the manuscript.

## Funding

The authors have nothing to report.

## Conflicts of Interest

A.D.T. and A.F. declare a conflicts of interest as consultants with Coya Therapeutics Inc. S.H.A. declares a conflicts of interest as the scientific advisory board chair of Coya Therapeutics Inc. The remaining authors have no conflicts of interest.

## Supporting information


**Supporting Information:** acn370381‐sup‐0001‐Supinfo.docx.

## Data Availability

The relevant data are presented in the manuscript. Statistical analyses used in this study are available. Supporting Informations cited in this manuscript are available.
