# Supplementary material for: Longitudinal Assessment of Biomarkers in ALS: Discriminative Biomarkers for Disease Progression and Survival
Source: Ann Clin Transl Neurol. 2026 Apr 6:10.1002/acn3.70381. Online ahead of print. doi: 10.1002/acn3.70381 (PMC13394509; doi:10.1002/acn3.70381)
Supplement: Supplementary file 1 — Supporting Information: acn370381‐sup‐0001‐Supinfo.docx. [file ACN3-9999-0-s001.docx]

Supplemental Materials

Comparing the three biomarkers individually, HNE consistently generated the highest AUCs (ranging from 0.78 to 0.80) among the individual biomarkers, indicating a better ability to predict poor survival status across all survival status thresholds. On the other hand, NfL generated the lowest AUCs (ranging from 0.65 to 0.66), which was lower than 0.70 and may suggest a limited predictive ability.

Furthermore, the difference in AUC among all potential combinations of the three biomarkers was statistically significant, where the AUC of “LBP & HNE & NfL” generated the highest AUCs among the combinations (ranging from 0.84 to 0.87).

**Table 2. Binary Survival status based on different survival month thresholds**

| **Biomarker** | **Optimal  Cut point** | **Sensitivity** | **Specificity** | **Index** | **AUC** | **p-value** |
| --- | --- | --- | --- | --- | --- | --- |
| **Median survival month (<27.5)** | | | | | | <0.01 |
| **LBP** |  |  |  |  | 0.73 (0.63 to 0.83) |  |
| Liu | 0.44 | 0.80 | 0.58 | 0.46 | 0.69 |  |
| Youden | 0.44 | 0.80 | 0.58 | 0.38 | 0.69 |  |
| **HNE** |  |  |  |  | 0.78 (0.69 to 0.87) |  |
| Liu | 0.56 | 0.62 | 0.92 | 0.57 | 0.77 |  |
| Youden | 0.56 | 0.62 | 0.92 | 0.54 | 0.77 |  |
| **NfL** |  |  |  |  | 0.65 (0.55 to 0.76) |  |
| Liu | 0.43 | 0.70 | 0.52 | 0.36 | 0.61 |  |
| Youden | 0.63 | 0.32 | 0.92 | 0.24 | 0.62 |  |
| **LBP & HNE** |  |  |  |  | 0.81 (0.73 to 0.90) |  |
| Liu | 0.55 | 0.62 | 0.92 | 0.57 | 0.77 |  |
| Youden | NA | NA | NA | NA | NA |  |
| **LBP & NfL** |  |  |  |  | 0.76 (0.67 to 0.86) |  |
| Liu | 0.49 | 0.72 | 0.74 | 0.53 | 0.73 |  |
| Youden | NA | NA | NA | NA | NA |  |
| **HNE & NfL** |  |  |  |  | 0.80 (0.72 to 0.89) |  |
| Liu | 0.48 | 0.68 | 0.80 | 0.54 | 0.74 |  |
| Youden | 0.63 | 0.58 | 0.92 | 0.50 | 0.75 |  |
| **LBP & HNE & NfL** |  |  |  |  | 0.84 (0.76 to 0.92) |  |
| Liu | 0.37 | 0.86 | 0.74 | 0.64 | 0.80 |  |
| Youden | 0.37 | 0.86 | 0.74 | 0.60 | 0.80 |  |
| **Mean survival month (<33.33)** | | | | | | <0.01 |
| **LBP** |  |  |  |  | 0.75 (0.65 to 0.85) |  |
| Liu | 0.55 | 0.78 | 0.63 | 0.49 | 0.71 |  |
| Youden | 0.55 | 0.78 | 0.63 | 0.41 | 0.71 |  |
| **HNE** |  |  |  |  | 0.78 (0.70 to 0.87) |  |
| Liu | 0.53 | 0.73 | 0.76 | 0.55 | 0.74 |  |
| Youden | 0.53 | 0.73 | 0.76 | 0.49 | 0.74 |  |
| **NfL** |  |  |  |  | 0.66 (0.55 to 0.76) |  |
| Liu | 0.53 | 0.68 | 0.54 | 0.37 | 0.61 |  |
| Youden | 0.72 | 0.32 | 0.98 | 0.30 | 0.65 |  |
| **LBP & HNE** |  |  |  |  | 0.84 (0.76 to 0.91) |  |
| Liu | 0.63 | 0.63 | 0.88 | 0.55 | 0.75 |  |
| Youden | 0.63 | 0.63 | 0.88 | 0.51 | 0.75 |  |
| **LBP & NfL** |  |  |  |  | 0.79 (0.70 to 0.87) |  |
| Liu | 0.61 | 0.68 | 0.78 | 0.53 | 0.73 |  |
| Youden | 0.61 | 0.68 | 0.78 | 0.46 | 0.73 |  |
| **HNE & NfL** |  |  |  |  | 0.80 (0.72 to 0.89) |  |
| Liu | 0.63 | 0.64 | 0.90 | 0.58 | 0.77 |  |
| Youden | 0.63 | 0.64 | 0.90 | 0.55 | 0.77 |  |
| **LBP & HNE & NfL** |  |  |  |  | 0.86 (0.79 to 0.93) |  |
| Liu | 0.51 | 0.81 | 0.83 | 0.67 | 0.82 |  |
| Youden | 0.51 | 0.81 | 0.83 | 0.64 | 0.82 |  |
| **3-Year survival month (<36)** | | | | | | <0.01 |
| **LBP** |  |  |  |  | 0.76 (0.67 to 0.86) |  |
| Liu | 0.67 | 0.68 | 0.74 | 0.50 | 0.71 |  |
| Youden | 0.67 | 0.68 | 0.74 | 0.42 | 0.71 |  |
| **HNE** |  |  |  |  | 0.80 (0.71 to 0.88) |  |
| Liu | 0.61 | 0.71 | 0.80 | 0.57 | 0.75 |  |
| Youden | 0.72 | 0.63 | 0.89 | 0.52 | 0.76 |  |
| **NfL** |  |  |  |  | 0.65 (0.54 to 0.76) |  |
| Liu | 0.67 | 0.49 | 0.74 | 0.36 | 0.62 |  |
| Youden | 0.76 | 0.34 | 0.94 | 0.28 | 0.64 |  |
| **LBP & HNE** |  |  |  |  | 0.85 (0.78 to 0.93) |  |
| Liu | 0.69 | 0.68 | 0.89 | 0.61 | 0.78 |  |
| Youden | 0.69 | 0.68 | 0.89 | 0.56 | 0.78 |  |
| **LBP & NfL** |  |  |  |  | 0.79 (0.70 to 0.88) |  |
| Liu | 0.64 | 0.71 | 0.80 | 0.57 | 0.75 |  |
| Youden | 0.64 | 0.71 | 0.80 | 0.51 | 0.75 |  |
| **HNE & NfL** |  |  |  |  | 0.81 (0.73 to 0.90) |  |
| Liu | 0.70 | 0.65 | 0.94 | 0.61 | 0.79 |  |
| Youden | 0.70 | 0.65 | 0.94 | 0.59 | 0.79 |  |
| **LBP & HNE & NfL** |  |  |  |  | 0.87 (0.80 to 0.94) |  |
| Liu | 0.57 | 0.85 | 0.83 | 0.71 | 0.84 |  |
| Youden | 0.57 | 0.85 | 0.83 | 0.68 | 0.84 |  |

Comparing the biomarkers respectively, HNE generated the highest AUC among the three biomarkers for Median (AUC=0.77) and Mean (AUC=0.83) Progression rate thresholds, where LBP generated the highest AUC based on the Q3 Progression rate threshold (AUC=0.83). Across the individual biomarker thresholds, NfL consistently generated the lowest AUC.

Furthermore, the difference in AUC among all potential combinations of the three biomarkers was statistically significant, where the AUC of “LBP & HNE & NfL” generated the highest AUCs among the combinations (ranging from 0.78 to 0.89).

**Table 3. Binary Progression status based on different Progression rate thresholds**

| **Biomarker** | **Optimal  Cut point** | **Sensitivity** | **Specificity** | **Index** | **AUC** | **p-value** |
| --- | --- | --- | --- | --- | --- | --- |
| **Median Progression rate (≥1.05)** | | | | | | 0.01 |
| **LBP** |  |  |  |  | 0.73 (0.63 to 0.83) |  |
| Liu | 0.58 | 0.53 | 0.88 | 0.47 | 0.70 |  |
| Youden | 0.58 | 0.53 | 0.88 | 0.41 | 0.70 |  |
| **HNE** |  |  |  |  | 0.77 (0.68 to 0.86) |  |
| Liu | 0.43 | 0.75 | 0.69 | 0.52 | 0.72 |  |
| Youden | 0.43 | 0.75 | 0.69 | 0.44 | 0.72 |  |
| **NfL** |  |  |  |  | 0.61 (0.50 to 0.73) |  |
| Liu | 0.49 | 0.65 | 0.61 | 0.40 | 0.63 |  |
| Youden | 0.49 | 0.65 | 0.61 | 0.26 | 0.63 |  |
| **LBP & HNE** |  |  |  |  | 0.78 (0.69 to 0.88) |  |
| Liu | 0.49 | 0.67 | 0.88 | 0.59 | 0.77 |  |
| Youden | 0.53 | 0.63 | 0.92 | 0.55 | 0.77 |  |
| **LBP & NfL** |  |  |  |  | 0.74 (0.64 to 0.84) |  |
| Liu | 0.50 | 0.75 | 0.65 | 0.49 | 0.70 |  |
| Youden | 0.58 | 0.57 | 0.84 | 0.41 | 0.70 |  |
| **HNE & NfL** |  |  |  |  | 0.77 (0.68 to 0.86) |  |
| Liu | 0.41 | 0.78 | 0.67 | 0.50 | 0.73 |  |
| Youden | 0.41 | 0.78 | 0.67 | 0.46 | 0.73 |  |
| **LBP & HNE & NfL** |  |  |  |  | 0.78 (0.69 to 0.88) |  |
| Liu | 0.48 | 0.67 | 0.88 | 0.59 | 0.77 |  |
| Youden | 0.53 | 0.63 | 0.92 | 0.55 | 0.77 |  |
| **Mean Progression rate (≥1.09)** | | | | | | <0.01 |
| **LBP** |  |  |  |  | 0.74 (0.63 to 0.84) |  |
| Liu | 0.48 | 0.60 | 0.80 | 0.48 | 0.70 |  |
| Youden | 0.51 | 0.56 | 0.85 | 0.41 | 0.71 |  |
| **HNE** |  |  |  |  | 0.83 (0.75 to 0.91) |  |
| Liu | 0.34 | 0.84 | 0.73 | 0.61 | 0.79 |  |
| Youden | 0.34 | 0.84 | 0.73 | 0.57 | 0.79 |  |
| **NfL** |  |  |  |  | 0.61 (0.50 to 0.72) |  |
| Liu | 0.45 | 0.53 | 0.69 | 0.37 | 0.61 |  |
| Youden | 0.45 | 0.53 | 0.69 | 0.22 | 0.61 |  |
| **LBP & HNE** |  |  |  |  | 0.83 (0.75 to 0.92) |  |
| Liu | 0.45 | 0.71 | 0.91 |  | 0.81 |  |
| Youden | 0.45 | 0.71 | 0.91 | 0.62 | 0.81 |  |
| **LBP & NfL** |  |  |  |  | 0.74 (0.64 to 0.84) |  |
| Liu | 0.49 | 0.62 | 0.80 |  | 0.71 |  |
| Youden | 0.49 | 0.62 | 0.80 | 0.42 | 0.71 |  |
| **HNE & NfL** |  |  |  |  | 0.83 (0.75 to 0.91) |  |
| Liu | 0.34 | 0.84 | 0.73 |  | 0.79 |  |
| Youden | 0.34 | 0.84 | 0.73 | 0.57 | 0.79 |  |
| **LBP & HNE & NfL** |  |  |  |  | 0.84 (0.75 to 0.92) |  |
| Liu | 0.39 | 0.78 | 0.85 |  | 0.82 |  |
| Youden | 0.39 | 0.78 | 0.85 | 0.63 | 0.82 |  |
| **Q3 Progression rate (≥1.53)** | | | | | | <0.01 |
| **LBP** |  |  |  |  | 0.83 (0.72 to 0.93) |  |
| Liu | 0.16 | 0.92 | 0.61 | 0.56 | 0.77 |  |
| Youden | 0.55 | 0.56 | 1.00 | 0.56 | 0.78 |  |
| **HNE** |  |  |  |  | 0.81 (0.71 to 0.91) |  |
| Liu | 0.21 | 0.84 | 0.73 | 0.61 | 0.79 |  |
| Youden | 0.21 | 0.84 | 0.73 | 0.57 | 0.79 |  |
| **NfL** |  |  |  |  | 0.77 (0.66 to 0.88) |  |
| Liu | 0.31 | 0.64 | 0.81 | 0.52 | 0.73 |  |
| Youden | 0.31 | 0.64 | 0.81 | 0.45 | 0.73 |  |
| **LBP & HNE** |  |  |  |  | 0.85 (0.76 to 0.95) |  |
| Liu | 0.19 | 0.80 | 0.76 | 0.61 | 0.78 |  |
| Youden | 0.59 | 0.60 | 0.99 | 0.59 | 0.79 |  |
| **LBP & NfL** |  |  |  |  | 0.88 (0.80 to 0.97) |  |
| Liu | 0.23 | 0.80 | 0.85 | 0.68 | 0.83 |  |
| Youden | 0.23 | 0.80 | 0.85 | 0.65 | 0.83 |  |
| **HNE & NfL** |  |  |  |  | 0.83 (0.75 to 0.92) |  |
| Liu | 0.23 | 0.84 | 0.76 | 0.64 | 0.80 |  |
| Youden | 0.23 | 0.84 | 0.76 | 0.60 | 0.80 |  |
| **LBP & HNE & NfL** |  |  |  |  | 0.89 (0.81 to 0.97) |  |
| Liu | 0.24 | 0.80 | 0.81 | 0.65 | 0.81 |  |
| Youden | 0.42 | 0.68 | 0.95 | 0.63 | 0.81 |  |

Comparing the individual biomarkers across thresholds, LBP consistently generated the highest AUCs (ranging from 0.54 to 0.58) among the three biomarkers, whereas HNE and NfL generated lower AUCs, and the difference in AUC between biomarkers was not statistically significant. However, all the AUCs of the biomarkers were lower than 0.70, indicating limited ability in predicting poor physical performance based on the ALSFR-S score.

Furthermore, no statistically significant difference in AUC among all potential combinations of the three biomarkers was found.

**Appendix Table 1. Binary ALS-FRS status based on different ALS-FRS thresholds**

| **Biomarker** | **Optimal  Cut point** | **Sensitivity** | **Specificity** | **Index** | **AUC** | **p-value** |
| --- | --- | --- | --- | --- | --- | --- |
| **Median ALS-FRS score (<37)** | | | | | | 0.60 |
| **LBP** |  |  |  |  | 0.54 (0.43 to 0.65) |  |
| Liu | 0.47 | 0.68 | 0.47 |  | 0.58 |  |
| Youden | 0.47 | 0.70 | 0.45 | 0.10 | 0.58 |  |
| **HNE** |  |  |  |  | 0.51 (0.40 to 0.63) |  |
| Liu | 0.48 | 0.45 | 0.64 |  | 0.54 |  |
| Youden | 0.48 | 0.30 | 0.79 | 0.09 | 0.55 |  |
| **NfL** |  |  |  |  | 0.49 (0.38 to 0.61) |  |
| Liu | 0.47 | 0.60 | 0.49 |  | 0.54 |  |
| Youden | 0.48 | 0.13 | 0.96 | 0.09 | 0.54 |  |
| **LBP & HNE** |  |  |  |  | 0.52 (0.41 to 0.64) |  |
| Liu | NA | NA | NA | NA | NA |  |
| Youden | 0.46 | 0.81 | 0.34 | 0.09 | 0.57 |  |
| **LBP & NfL** |  |  |  |  | 0.53 (0.42 to 0.65) |  |
| Liu | 0.47 | 0.68 | 0.45 |  | 0.57 |  |
| Youden | 0.46 | 0.85 | 0.28 | 0.13 | 0.57 |  |
| **HNE & NfL** |  |  |  |  | 0.52 (0.41 to 0.64) |  |
| Liu | 0.48 | 0.51 | 0.60 |  | 0.56 |  |
| Youden | 0.48 | 0.36 | 0.75 | 0.12 | 0.56 |  |
| **LBP & HNE & NfL** |  |  |  |  | 0.52 (0.41 to 0.64) |  |
| Liu | NA | NA | NA | NA | NA |  |
| Youden | 0.46 | 0.81 | 0.34 | 0.15 | 0.57 |  |
| **Mean ALS-FRS score (<36)** | | | | | | 0.70 |
| **LBP** |  |  |  |  | 0.55 (0.43 to 0.67) |  |
| Liu | 0.41 | 0.51 | 0.68 | 0.35 | 0.60 |  |
| Youden | 0.41 | 0.51 | 0.68 | 0.19 | 0.64 |  |
| **HNE** |  |  |  |  | 0.53 (0.41 to 0.65) |  |
| Liu | NA | NA | NA | NA | NA |  |
| Youden | 0.42 | 0.32 | 0.80 | 0.11 | 0.56 |  |
| **NfL** |  |  |  |  | 0.54 (0.42 to 0.65) |  |
| Liu | 0.40 | 0.51 | 0.59 | 0.30 | 0.55 |  |
| Youden | 0.38 | 0.88 | 0.24 | 0.12 | 0.56 |  |
| **LBP & HNE** |  |  |  |  | 0.53 (0.40 to 0.65) |  |
| Liu | 0.42 | 0.41 | 0.80 | 0.33 | 0.61 |  |
| Youden | 0.43 | 0.39 | 0.83 | 0.22 | 0.61 |  |
| **LBP & NfL** |  |  |  |  | 0.55 (0.43 to 0.67) |  |
| Liu | 0.42 | 0.39 | 0.75 | 0.29 | 0.57 |  |
| Youden | 0.45 | 0.29 | 0.90 | 0.19 | 0.60 |  |
| **HNE & NfL** |  |  |  |  | 0.54 (0.42 to 0.66) |  |
| Liu | 0.42 | 0.46 | 0.69 | 0.32 | 0.58 |  |
| Youden | 0.42 | 0.46 | 0.69 | 0.16 | 0.58 |  |
| **LBP & HNE & NfL** |  |  |  |  | 0.54 (0.42 to 0.66) |  |
| Liu | 0.41 | 0.46 | 0.66 | 0.30 | 0.56 |  |
| Youden | 0.45 | 0.29 | 0.90 | 0.19 | 0.60 |  |
| **Q3 ALS-FRS score (<41)** | | | | | | 0.47 |
| **LBP** |  |  |  |  | 0.58 (0.46 to 0.70) |  |
| Liu | 0.75 | 0.47 | 0.81 | 0.38 | 0.64 |  |
| Youden | 0.75 | 0.47 | 0.81 | 0.28 | 0.64 |  |
| **HNE** |  |  |  |  | 0.53 (0.39 to 0.66) |  |
| Liu | 0.74 | 0.57 | 0.54 | 0.31 | 0.55 |  |
| Youden | 0.73 | 0.70 | 0.42 | 0.13 | 0.56 |  |
| **NfL** |  |  |  |  | 0.52 (0.40 to 0.64) |  |
| Liu | 0.74 | 0.49 | 0.65 | 0.32 | 0.57 |  |
| Youden | 0.77 | 0.20 | 0.96 | 0.16 | 0.58 |  |
| **LBP & HNE** |  |  |  |  | 0.58 (0.46 to 0.70) |  |
| Liu | 0.73 | 0.66 | 0.54 | 0.36 | 0.60 |  |
| Youden | 0.71 | 0.74 | 0.46 | 0.21 | 0.60 |  |
| **LBP & NfL** |  |  |  |  | 0.57 (0.44 to 0.69) |  |
| Liu | 0.74 | 0.51 | 0.62 | 0.32 | 0.56 |  |
| Youden | 0.76 | 0.35 | 0.85 | 0.20 | 0.60 |  |
| **HNE & NfL** |  |  |  |  | 0.52 (0.40 to 0.65) |  |
| Liu | 0.73 | 0.53 | 0.62 | 0.33 | 0.57 |  |
| Youden | 0.77 | 0.22 | 0.96 | 0.18 | 0.59 |  |
| **LBP & HNE & NfL** |  |  |  |  | 0.59 (0.47 to 0.71) |  |
| Liu | 0.72 | 0.69 | 0.54 | 0.37 | 0.61 |  |
| Youden | 0.72 | 0.69 | 0.54 | 0.23 | 0.61 |  |

Keeping all other biomarkers constant, HNE generated the strongest association with survival, where a one µg/ml increase in HNE was significantly associated with a 27% increase in the hazard of death. We also found that a one µg/ml increase in LBP was significantly associated with a 5% increase in the hazard of death. However, no statistically significant association was found between NfL and survival after accounting for the other biomarkers.

**Table 3A. Hazard ratio of survival associated with the biomarkers.**

| **Biomarker** | **Hazard Ratio** | **95% Confidence Interval** |
| --- | --- | --- |
| **LBP** | 1.05 | 1.02 to 1.08 |
| **HNE** | 1.27 | 1.19 to 1.37 |
| **NfL** | 1.00 | 1.00 to 1.01 |

All three biomarkers are significantly associated with the hazard of death in the univariable Cox model, where a unit increase in the biomarker is associated with a 9%, 31%, and 1% of the hazard of death for LBP, HNE, and NfL, respectively.

**Table 3B. Hazard ratio of survival associated with LBP**

| **Biomarker** | **Hazard Ratio** | **95% Confidence Interval** |
| --- | --- | --- |
| **LBP** | 1.09 | 1.06 to 1.12 |

**Table 3C. Hazard ratio of survival associated with HNE**

| **Biomarker** | **Hazard Ratio** | **95% Confidence Interval** |
| --- | --- | --- |
| **HNE** | 1.31 | 1.23 to 1.40 |

**Table 3D. Hazard ratio of survival associated with NfL**

| **Biomarker** | **Hazard Ratio** | **95% Confidence Interval** |
| --- | --- | --- |
| **NfL** | 1.01 | 1.004 to 1.01 |

Keeping all other biomarkers constant, a one µg/ml increase in LBP was significantly associated with a 0.0314 increase in progression rate (ALSFRS points/month); and a one µg/ml increase in HNE was significantly associated with a 0.0414 increase in progression rate. On the other hand, no statistically significant association with progression rate was found for NfL while accounting for all the biomarkers.

**Table 4A. Association of Progression rate and the biomarkers.**

| **Biomarker** | **Estimate** | **SE** | **p-value** |
| --- | --- | --- | --- |
| **LBP** | 0.0314 | 0.0056 | <0.01 |
| **HNE** | 0.0414 | 0.0104 | <0.01 |
| **NfL** | 0.0005 | 0.0008 | 0.51 |

All three biomarkers are significantly associated with the progression rate (ALSFRS points/month) in the univariable regression model, where a unit increase in the biomarker is associated with a 0.0421, 0.0665, and 0.0031 of the progression rates for LBP, HNE, and NfL, respectively.

**Table 4B. Association of Progression rate and the biomarkers.**

| **Biomarker** | **Estimate** | **SE** | **p-value** |
| --- | --- | --- | --- |
| **LBP** | 0.0421 | 0.0056 | <0.01 |

**Table 4C. Association of Progression rate and the biomarkers.**

| **Biomarker** | **Estimate** | **SE** | **p-value** |
| --- | --- | --- | --- |
| **HNE** | 0.0665 | 0.0102 | <0.01 |

**Table 4D. Association of Progression rate and the biomarkers.**

| **Biomarker** | **Estimate** | **SE** | **p-value** |
| --- | --- | --- | --- |
| **NfL** | 0.0031 | 0.0009 | <0.01 |

**Appendix Table 2. Descriptive analysis of the ALS-FRS.**

| **Characteristics** | **Median/Mean** | **IQR/SD** | **p-value** |
| --- | --- | --- | --- |
| ALS-FRS | 37.00 | [32.00, 41.00] | <0.01 |

Keeping all other biomarkers constant, HNE was significantly associated with a higher ALSFRS-R score, where one µg/ml increase in HNE was significantly associated with a 0.55 unit increase in ALSFRS-R score. However, no statistically significant association with ALSFRS-R score was found for LBP and NfL while accounting for all the biomarkers.

**Appendix Table 3A. Association of ALSFRS-R score and the biomarkers.**

| **Biomarker** | **Estimate** | **SE** | **p-value** |
| --- | --- | --- | --- |
| **LBP** | -0.20 | 0.13 | 0.14 |
| **HNE** | 0.55 | 0.24 | 0.03 |
| **NfL** | -0.01 | 0.02 | 0.53 |

No evidence that the biomarkers are all associated with ALSFRS-R score in the univariable regression model.

**Appendix Table 3B. Association of ALSFRS-R score and the biomarkers.**

| **Biomarker** | **Estimate** | **SE** | **p-value** |
| --- | --- | --- | --- |
| **LBP** | -0.0925 | 0.1216 | 0.449 |

**Appendix Table 3C. Association of ALSFRS-R score and the biomarkers.**

| **Biomarker** | **Estimate** | **SE** | **p-value** |
| --- | --- | --- | --- |
| **HNE** | 0.3461 | 0.2108 | 0.104 |

**Appendix Table 3D. Association of ALSFRS-R score and the biomarkers.**

| **Biomarker** | **Estimate** | **SE** | **p-value** |
| --- | --- | --- | --- |
| **NfL** | -0.0018 | 0.0170 | 0.92 |

**AUC internal validation with bootstrapping: Mean Survival month**

| Models | Apparent AUC | Apparent 95% CI | Mean Optimism | Mean Optimism 95% CI | Optimism-adjusted AUC |
| --- | --- | --- | --- | --- | --- |
| LBP | 0.75 | 0.66 to 0.85 | -0.0014 | -0.0036 to 0.0008 | 0.75 |
| HNE | 0.79 | 0.69 to 0.87 | -0.0003 | -0.0023 to 0.0017 | 0.79 |
| NfL | 0.66 | 0.55 to 0.76 | 0.0012 | -0.0012 to 0.0035 | 0.66 |
| Lbp+HNE | 0.84 | 0.76 to 0.91 | 0.0082 | 0.0064 to 0.0099 | 0.83 |
| LBP+NfL | 0.79 | 0.70 to 0.87 | 0.0087 | 0.0068 to 0.0107 | 0.78 |
| HNE+NfL | 0.81 | 0.72 to 0.89 | 0.0087 | 0.0069 to 0.0106 | 0.80 |
| LBP+HNE+NfL | 0.86 | 0.78 to 0.93 | 0.0146 | 0.0129 to 0.0162 | 0.85 |

**AUC internal validation with bootstrapping: Mean Progression rate**

| Models | Apparent AUC | Apparent 95% CI | Mean Optimism | Mean Optimism 95% CI | Optimism-adjusted AUC |
| --- | --- | --- | --- | --- | --- |
| LBP | 0.74 | 0.63 to 0.83 | 0.0005 | -0.0017 to 0.0028 | 0.74 |
| HNE | 0.83 | 0.74 to 0.91 | 0.0022 | 0.0004 to 0.0041 | 0.83 |
| NfL | 0.61 | 0.51 to 0.72 | 0.0060 | 0.0035 to 0.0086 | 0.60 |
| Lbp+HNE | 0.83 | 0.75 to 0.92 | 0.0102 | 0.0084 to 0.0121 | 0.82 |
| LBP+NfL | 0.74 | 0.65 to 0.84 | 0.0133 | 0.0111 to 0.0155 | 0.73 |
| HNE+NfL | 0.83 | 0.75 to 0.91 | 0.0100 | 0.0082 to 0.0118 | 0.82 |
| LBP+HNE+NfL | 0.84 | 0.76 to 0.92 | 0.0178 | 0.0160 to 0.0197 | 0.82 |
